# Synthesis, Photo- and Electroluminescence of New Polyfluorene Copolymers Containing Dicyanostilbene and 9,10-Dicyanophenanthrene in the Main Chain

**DOI:** 10.3390/ma16165592

**Published:** 2023-08-12

**Authors:** Anton A. Yakimanskiy, Ksenia I. Kaskevich, Elena V. Zhukova, Ivan A. Berezin, Larisa S. Litvinova, Tatiana G. Chulkova, Dmitriy A. Lypenko, Artem V. Dmitriev, Sergey I. Pozin, Natalia V. Nekrasova, Felix N. Tomilin, Daria A. Ivanova, Alexander V. Yakimansky

**Affiliations:** 1 Institute of Macromolecular Compounds RAS, Bolshoi Prospect of Vasilyevsky Island 31, St. Petersburg 199004, Russia; yakimanskii@gmail.com (A.A.Y.); kaskevich-ksenia@yandex.ru (K.I.K.); zukovaev@mail.ru (E.V.Z.); bereziv@mail.ru (I.A.B.); larissa_litvinova@hotmail.com (L.S.L.); tgc@mail.ru (T.G.C.); 2 A.N. Frumkin Institute of Physical Chemistry and Electrochemistry RAS, Leninskiy Prospect 31, bld.4, Moscow 119071, Russia; dalypenko@gmail.com (D.A.L.); oleduff@mail.ru (A.V.D.); sergip74@gmail.com (S.I.P.); natalianek@yandex.ru (N.V.N.); 3 Kirensky Institute of Physics, Federal Research Center KSC SB RAS, Krasnoyarsk 660036, Russia; felixnt@gmail.com; 4 School of Non-Ferrous Metals and Material Science, Siberian Federal University, Krasnoyarsk 660041, Russia; daha-ha@mail.ru

**Keywords:** copolyfluorene, phenanthrene-9,10-dicarbonitrile, α,α′-dicyanostilbene, luminescence, OLEDs

## Abstract

Using palladium-catalyzed Suzuki polycondensation, we synthesized new light-emitting fluorene copolymers containing the dicyano derivatives of stilbene and phenanthrene and characterized them by gel permeation chromatography, UV-vis absorption spectroscopy, spectrofluorimetry, and cyclic voltammetry. The photoluminescence spectra of the synthesized polymers show significant energy transfer from the fluorene segments to the dicyanostilbene and 9,10-dicyanophenanthrene units, which is in agreement with the data of theoretical calculations. OLEDs based on these polymers were fabricated with an ITO/PEDOT-PSS (35 nm)/p-TPD (30 nm)/PVK (5 nm)/light emitting layer (70–75 nm)/PF-PO (20 nm)/LiF (1 nm)/Al (80 nm) configuration. Examination of their electroluminescence revealed that copolymers of fluorene with dicyanostilbene show yellow-green luminescence, while polymers with 9,10-dicyanophenanthrene have a greenish-blue emission. The 9,10-dicyanophenanthrene units have a more rigid structure compared to dicyanostilbene and, in OLEDs based on them, an increase in maximum brightness is observed with an increase in the content of the additive to the polymer chain. In particular, the device using fluorene copolymer with 9,10-dicyanophenanthrene (2.5 mol%) exhibited a maximum brightness of 9230 cd/m^2^ and a maximum current efficiency of 3.33 cd/A.

## 1. Introduction

Among the π-conjugated polymers, polyfluorenes (PFs) and fluorene-based copolymers are widely studied due to their exceptional optoelectronic properties [1,2,3,4,5]. An important driving force for such research is the preparation of new polymeric materials for organic light-emitting devices (OLEDs), which can be used in ultrathin and flexible screens for computers, televisions, mobile phones, smartwatches, wearable healthcare systems, and augmented reality/virtual reality technology. In the light-emitting polymer film sandwiched between two electrodes, the injected holes and electrons migrate across the polymer layer and combine to form excitons, which then decay with photon emission. Depending upon the chemical structure of the emissive polymer, different colours can be obtained. One of the most important characteristics of copolyfluorenes is the possibility of the fine tuning of electrooptical properties due to functionalization with phosphor, donor–acceptor, charge-transport, and metal-binding fragments. Polymers based on fluorene units are distinguished by their high chemical and thermal stability, solubility in basic organic solvents (chloroform, toluene, THF, DMF), good film-forming properties, high luminescence quantum yield in solution and in coatings, and the possibility of their forming a liquid crystal phase.

In this work, we synthesized and investigated the spectral characteristics of six fluorene copolymers with the dicyano derivatives of stilbene and phenanthrene. Dicyanostilbenes and 9,10-dicyanophenanthrenes are luminescent compounds with aggregation-induced emission [6,7,8]. Such compounds generate highly efficient solid emission and have advanced practical applications. The effects of different dicyano derivatives on the photophysical and electroluminescence spectral characteristics of copolyfluorenes are compared and discussed in this paper.

## 2. Materials and Methods

All reagents and starting materials were purchased from commercial suppliers and used without further purification. Toluene (99%, Sigma-Aldrich, St. Louis, MI, USA) was distilled twice over sodium. Deionized water was obtained from a water purification system (RiOs-DI 3 Smart, Millipore, Merck, Darmstadt, Germany). All other solvents were purified by standard methods. Silica gel 60 (0.2–0.5 mm, Macherey-Nagel, Duren, Germany) underwent column chromatography and QuadraSil™ metal scavenger (20–100 micron, Alfa-Aesar, Ward Hill, MA, USA) was applied to remove Pd residuals.

The polycondensation reactions were performed in the CEM Discover LabMate single-mode microwave reactor (CEM Corporation, Matthews, NC, USA) at a radiation frequency of 2.45 GHz and a maximum generator power of 300 W. The temperature of synthesis was controlled using an infrared sensor placed under the reaction vessel. The reaction parameters (temperature, power, time, stirring rate) were set manually.

Polymer films were prepared on an Ossila spin coater and dried or heated in a UT-4620 drying chamber. The CPF films were formed by spin coating on the glass from polymers solutions in toluene (200–220 µL). The toluene solution concentration was 10 mg/mL.

NMR spectra were recorded on a Bruker AVANCE–400 SB (400 MHz) spectrometer at room temperature. The UV-visible absorption spectra were recorded on a Shimadzu UV-1900 spectrophotometer. Photoluminescence spectra were measured using an RF-6000 spectrofluorophotometer. For studying luminescence and absorption spectra in solution, a 0.02 mg/mL concentration of each corresponding CPF in chloroform was chosen. The absorption and emission spectra of the films were measured immediately before heating and then after exposure for 4 h in the drying chamber at 80 °C and a high ventilation mode. FT-IR spectra were recorded on a Shimadzu IR Affinity-1S spectrometer using a Quest single-reflection ATR accessory (Specac), KRS-5 prism, 7800–400 cm^−1^ range.

### 2.1. Synthesis of 2,3-Di(4-bromophenyl)fumarodinitrile and 3,6-Dibromophenathrene-9,10-dicarbonitrile

2,3-Di(4-bromophenyl)fumarodinitrile and 3,6-dibromophenanthrene-9,10-dicarbonitrile were synthesized according to procedure described previously [6,7,9,10,11]. The substances were purified by silica gel column chromatography (eluent mixture dichloromethane: hexane 7:3) and their structures were then verified by means of ^1^H NMR, FT-IR, and by melting point determination.

### 2.2. Synthesis of Copolyfluorenes (CPFs) [12]

The synthesis of CPFs was carried out in a 100 mL cylinder vessel for microwave reactor equipped with a reflux condenser and an adapter with two valves to operate in the argon/vacuum system and for introduction of solvents and solutions.

9,9-Dioctyl-9H-fluorene-2,7-diboronic acid bis(pinacol) ester and dibromo monomers were taken in the molar ratio of 1.025 to 1. Three different loads of 2,7-dibromo-9,9-dioctyl-9H-fluorene (135.7 mg (0.2475 mmol), 134.4 mg (0.2450 mmol), and 130.3 mg (0.2375 mmol), respectively) were complimented by comonomers of 2,3-di(4-bromophenyl)fumarodinitrile (0.97 mg (0.0025 mmol), 1.9 mg (0.0050 mmol), and 4.9 mg (0.0125 mmol)) or 3,6-dibromophenanthrene-9,10-dicarbonitrile (0.96 mg (0.0025 mmol), 1.9 mg (0.0050 mmol), and 4.9 mg (0.0125 mmol)) to result in 0.25 mmol of dibromo monomers loading.

In typical procedure, 9,9-dioctyl-9H-fluorene-2,7-diboronic acid bis(pinacol) ester (0.1647 g, 0.25625 mmol), dibromo monomers, and triphenylphosphine (5 mg, 0.019 mmol) were introduced in the reaction vessel along with stirring bar. Oxygen was removed from the system, and the (PPh_3_)_4_Pd catalyst (6 mg, 0.005 mmol, 1 mol%) was added in a glovebox chamber under argon atmosphere. The system was then evacuated again and purged with argon three times. Then, 2.5 mL of toluene, 2 mL of 2M K_2_CO_3_ solution, and methyltrioctylammonium chloride (Aliquat^®^ 336) (10 mg, 0.019 mmol) in 1 mL of toluene, all three initially bubbled with moderate argon flow for 1 h, were loaded through a reflux condenser into the reaction vessel.

The cylinder vessel with condenser was placed in a microwave reactor, and the reaction mixture was stirred at an average of 90 °C (radiation power 80 W) for 1.5 h. Then, the first end-capping reagent, phenylboronic acid pinacol ester (4 mg in 1 mL of toluene, 0.02 mmol), was added to the reaction mixture, and after heating for another 1 h, the second end-capping reagent, 4-bromophenyl ethyl ether (15 µL in 1 mL of toluene, 0.1 mmol), was added to the reaction mixture. Stirring and heating continued for another 1.5 h, and the resulting two-phase mixture was cooled down to room temperature and poured into excess of methanol to form a precipitate.

The resulting precipitate was washed with water twice, then with methanol, and dried on filter, redissolved in CHCl_3_, passed through a metal-scavenger silica gel-based small column, and reprecipitated in methanol. Low-molecular mass fractions of polymer were extracted with acetone in the Soxhlet apparatus (until the acetone solution became visually colorless, usually 12–14 h reflux). Then, the precipitate was dissolved in CHCl_3_, filtered through Chromafil^®^ Xtra PET-45/25 syringe filter, and, finally, reprecipitated in methanol. The yields of the CPFs after purification were 40–60%.

^1^H NMR and FT-IR spectra interpretation for the synthesized compounds are given in the Supplementary Materials, Table S1.

### 2.3. Determination of the Molar Masses of Synthesized Polymers

Weight-average molecular weight (M_w_), number-average molecular weight (M_n_), and molecular weight polydispersity ratio (M_w_/M_n_) of the samples were estimated by size exclusion chromatography (SEC). We used an Agilent Technologies 1260 Infinity liquid chromatograph (the Agilent 1260 Infinity Multi-Detector GPC/SEC System) equipped with three detectors: refractometric (DRI, laser wavelength 660 nm), viscometric (VS), and light scattering (LS, Rayleigh scattering angles 15° and 90°; laser wavelength 660 nm, power 50 mW). The detector temperature was 40 °C. The setup included a set of connected chromatographic columns: a PLgel 5 µm Guard 50 7.5 mm precolumn and two Agilent Technologies PLgel 5 µm MIXED-C, 300 7.5 mm columns. Their temperature was also 40 °C. THF distilled over KOH and stabilized by 2,6-di-tert-butyl-4-hydroxytoluene (0.02%) was used as the mobile phase (flow rate 1.0 mL/min). Sample injection was performed using an autosampler; the sample volume was 100 µL. Sample concentration did not exceed 2 mg/mL. Different combinations of detectors can be used for sample analysis: a single-detector SEC with the DRI detector (GPC), DRI-VS (VS, two-detector SEC), DRI-LS (LS, two-detector SEC), and DRI-VS-LS (TRIPLE, three-detector SEC).

Polystyrene standards (Agilent Technologies) were used for column calibration in the GPC mode (DRI detector) and for obtaining the universal calibration (combination of DRI and VS detectors). A three-detector SEC (DRI-VS-LS), as well as the DRI-LS combination, does not require calibration. SEC data were analyzed using Agilent GPC/SEC Software, version 1.2.

### 2.4. Atomic Force Microscopy (AFM)

The surface morphology of CPF layers prepared by spin-coating from a toluene solution was studied using an EnviroScope atomic force microscope with a Nanoscope-V controller (Veeco). For optimal interaction with the surface, the scanning mode was selected using cantilevers with different spring constants. It was checked that the static electric charge did not smooth the images.

Scanning was performed in tapping mode (repulsive regime). Standard cantilevers were used (force constant 10–40 N/m, resonance frequencies 150–300 kHz, curvature radius ≈ 10 nm).

### 2.5. Electroluminescence Studies

Initially, we considered three types of the light-emitting diodes, which consisted of the following layers: ITO/PEDOT-PSS (35 nm)/EML (70–75 nm)/PF-PO (20 nm)/LiF (1 nm)/Al (80 nm) for system I, ITO/PEDOT-PSS (35 nm)/TFB (30 nm)/EML (70–75 nm)/PF-PO (20 nm)/LiF (1 nm)/Al (80 nm) for system II, and ITO/PEDOT-PSS (35 nm)/*p*-TPD (30 nm)/PVK (5 nm)/EML (70–75 nm)/PF-PO (20 nm)/LiF (1 nm)/Al (80 nm) for system III. Preliminary studies of selected CPF sample (PFCN1) showed system III PLED to give the best results in terms of turn-on voltage and maximum brightness luminance. Therefore, electroluminescent properties of the CPFs were compared on the system III-type PLEDs.

To obtain a PLED sample, the thoroughly cleaned and treated by oxygen plasma glass plate with ITO layer was covered with PEDOT-PSS (Clevios™ P VP AI 4083, Heraeus) layer from its water solution by centrifugation. The plate was dried at 110 °C for 20 min. The next layers of *p*-TPD (8 mg/mL solution in chlorobenzene) and PVK (1.5 mg/mL solution in *m*-xylol) were processed at a centrifuge speed of 2000 rpm, followed by plate drying for another 20 min at 110 and 140 °C, respectively. The EML was formed by pouring CPF solution in toluene (10 mg/mL) at 2500 rpm. Therefore, plates were dried for 4 h at 80 °C (some samples were kept at 110 °C for another 20 min). Next, electron transporting layer PF-PO was prepared from ethanol solution (5 mg/mL) at 1500 rpm and dried for 3 h in argon atmosphere at 60 °C. Finally, electron layer (LiF) and cathode (Al) were deposited on a plate by thermal evaporation under vacuum (2 × 10^−6^ mbar). The electroluminescence (EL) spectra of PLEDs were recorded using an Avantes 2048 fiber-optic spectrofluorimeter (Apeldoorn, Netherlands). Voltage–current and voltage–brightness characteristics were recorded with a Keithley 2601 SourceMeter (Tektronix, Beaverton, OR, USA), a Keithley 485 picoampermeter, and a TKA-04/3 luxmeter-brightness meter (TKA Scientific Instruments, Saint Petersburg, Russia). Preparations of the PLED samples and the measurements of their spectral and optoelectronic characteristics were performed at room temperature in the argon atmosphere glovebox with maximum oxygen and moisture presence of 10 ppm. The absolute external quantum efficiency (EQE) of OLED was obtained by experimental setup utilizing an integrating sphere (VNIIOFI, Russia).

### 2.6. Cyclic Voltammetry (CVA)

HOMO and LUMO energy levels in thin layers of the CPFs were determined by cyclic voltammetry (CV) at a scan rate of 20 mV/s in 0.2 M solution of tetrabutylammonium hexafluorophosphate in acetonitrile, as described earlier [13]. A pseudo reference electrode Ag/AgNO3 was calibrated against ferrocene/ferricenium couple assuming its energy level to be −4.988 eV in acetonitrile [14].

### 2.7. Calculation Methods

**Figure 1:**
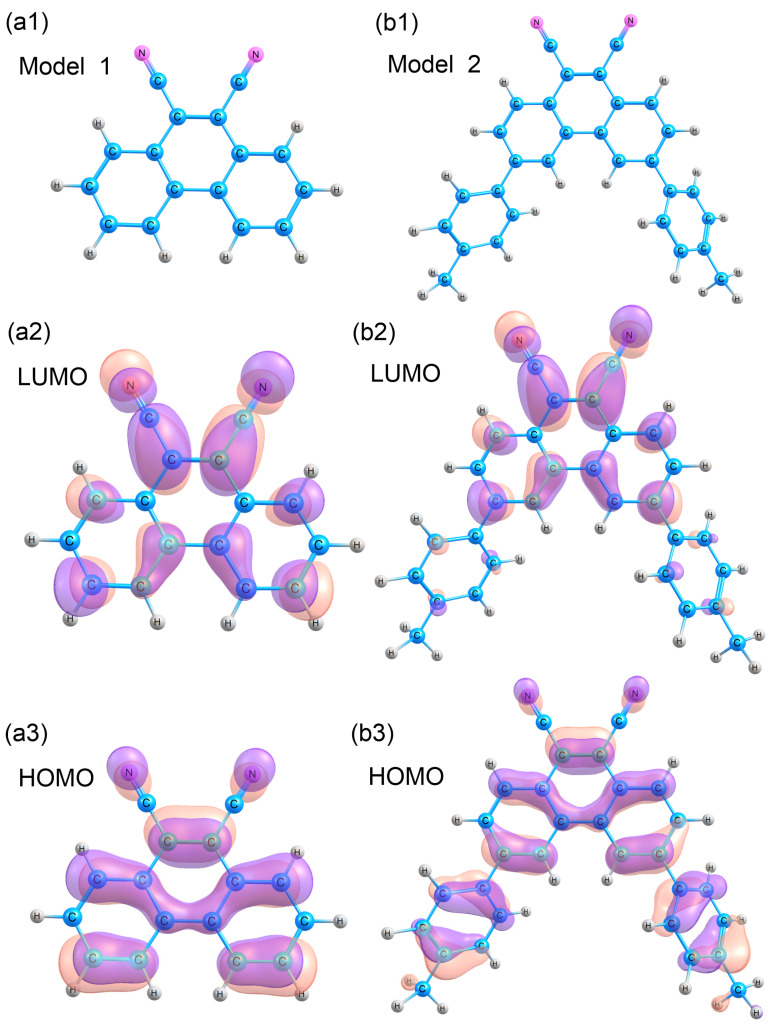
Models for the calculation of the phenanthrene-9,10-dicarbonitrile molecule. Phenanthrene-9,10-dicarbonitrile—model 1 (**a1**): isosurfaces of LUMO (**a2**) and HOMO (**a3**). 3,6-di(4-methylphenyl)phenanthrene-9,10-dicarbonitrile—model 2 (**b1**): isosurfaces of LUMO (**b2**) and HOMO (**b3**).

Quantum chemical calculations of the phenanthrene molecule were performed by density functional theory (DFT) within the hybrid B3LYP and CAM-B3LYP [15] functional with the cc-pVDZ basis. The SMD model [16] was used to account for the solvent (chloroform). Time-dependent density functional theory (TD-DFT) was used to calculate the absorption spectrum maxima of the molecules [17], to optimize the excited state geometries, and to calculate the luminescence spectrum maximum for the excited atomic structure. All calculations were carried out in the GAMESS program [18]. The course of the calculations was as follows: first, the effect of conjugation on the phenanthrene molecule was studied in the B3LYP method. For this purpose, phenanthrene-9,10-dicarbonitrile with hydrogen—model 1 (Figure 1(a1)), and with *p*-tolyl substituents in positions 3,6—model 2, (Figure 1(b1)) were optimized. The absorption spectrum was then calculated for the best model using the two B3LYP and CAM-B3LYP functionals. The second functional was used to check possible processes related to charge transfer.

## 3. Results and Discussion

### 3.1. Synthesis and Characterization of Copolyfluorenes

**Figure 2:**
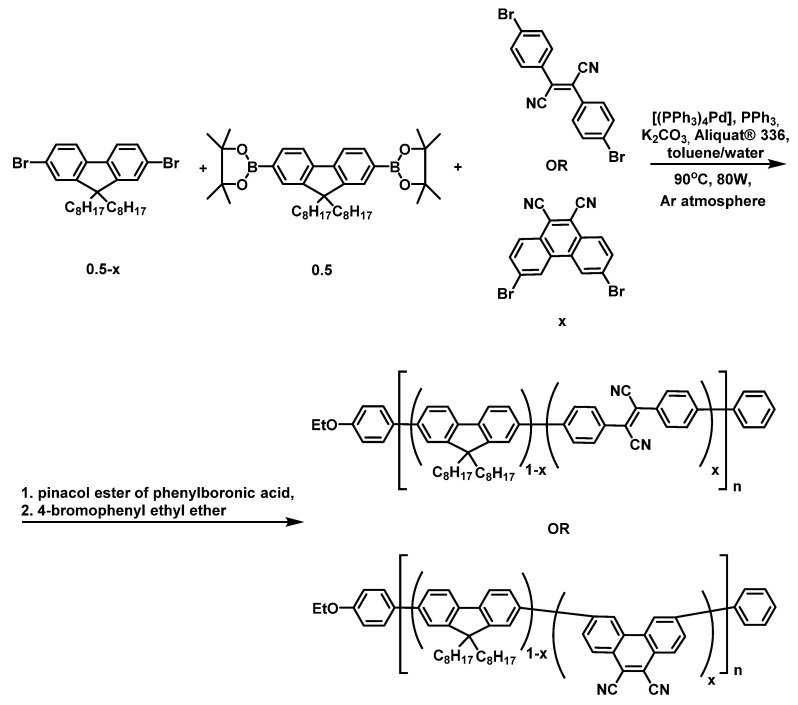
Synthesis of copolyfluorenes with dicyanostilbene (FFCNx) and dicyanophenanthrene (PFCNx) moieties.

The two types of copolyfluorenes (FFCNx and PFCNx) were synthesized via Suzuki−Miyaura cross-coupling reaction using 9,9-dioctyl-9H-fluorene-2,7-diboronic acid bis(pinacol) ester, 2,7-dibromo-9,9-dioctyl-9H-fluorene, and either 2,3-di(4-bromophenyl)fumarodinitrile or 3,6-dibromophenanthrene-9,10-dicarbonitrile under microwave irradiation (80 W) and an inert atmosphere at 90 °C (Figure 2). The reaction was catalyzed by (PPh_3_)_4_Pd(0) in the presence of PPh_3_, K_2_CO_3_, and Aliquat^®^ 336 (as a phase transfer catalyst) in the two-phase toluene/water media. The presence of terminal fragments in polymer chains is necessary for the removal of reactive end groups (Br and pinacol ester of boronic acid), which have a detrimental effect on the optoelectronic properties of the polymers. Therefore, pinacol ester of phenylboronic acid and 4-bromophenyl ethyl ether were introduced into the reaction mixture as end-capping reagents.

The molecular mass characteristics of copolyfluorenes are presented in Table 1.

The molecular masses of the resulting products depend on the type and amount of dicyano fragments. Two factors which can affect the molecular mass characteristics of the obtained CPFs are listed below. First, 2,3-di(4-bromophenyl)fumarodinitrile is introduced into the main chain without disturbing the linearity of the polymer structure, while the phenanthrene fragment twists the polymer chain. As a result, the MMs decrease with an increasing content of the dicyano moiety, and to a greater extent for PFCN than for FFCN.

Second, nitriles can deactivate catalytic particles by binding to Pd [19]. This can be the reason for the decrease in molecular masses observed with increasing comonomer loading.

^1^H NMR and IR spectral data for the synthesized compounds are given in the Supplementary Materials, Table S1, Figures S1 and S2. The ^1^H NMR spectra of all the synthesized copolyfluorenes contain the main signals of the aromatic protons of the fluorene fragment at 7.70–7.74 (m, 4H) and 7.86–7.88 (m, 2H) ppm and the protons of the octyl groups at 0.83–0.86 (m, 6H, CH_3_), 1.17–1.25 (m, 24H, CH_2_), and 1.75–2.50 (m, 4H, CH_2_) ppm. The signals of the dicyanostilbene and dicyanophenanthrene units at 7.60–8.12 ppm have a low intensity due to the low content of the corresponding comonomers, and partially overlap with the signals of the fluorene fragment. The signals at 7.00–7.60 ppm belong to the aromatic protons of the end-capping groups (phenyl and 4-ethoxyphenyl). Figure S2 shows the FTIR spectra of the polymers FFCNx and PFCNx. The CPFs absorb at 2920 and 2850 cm^−1^ due to C−H stretching vibrations, and at 1450 cm^−1^ due to the C=C_Ar_ stretching vibrations. The FTIR spectra of the CPFs exhibited no distinctive band for the cyano group due to the low content of the corresponding comonomer.

### 3.2. AFM Study of Copolyfluorene Films

**Figure 3:**
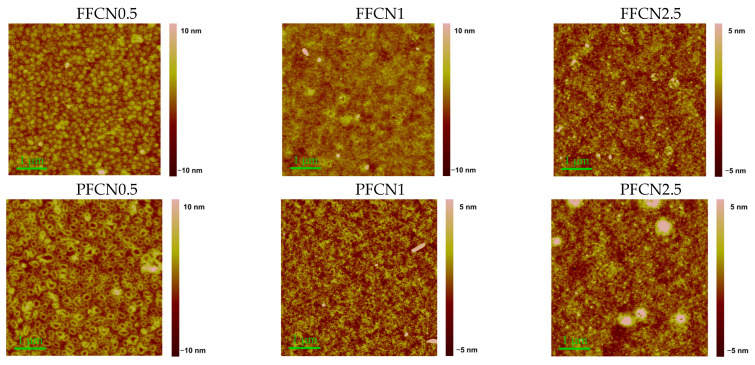
AFM scans of copolyfluorene films heated at 80 °C.

Atomic force microscopy (AFM) images allow for a comparison of the nanoscale ordering of the films. Figure 3 shows the AFM images of films heated at 80 °C. The topography of the films depends on the content of dicyano-substituted comonomer in the polymer chain, but practically does not depend on the type of dicyano-substituted comonomer.

The surface of FFCN0.5 and PFCN0.5 samples has a granular morphology with polygonal particles (granules) that are 2–6 nm thick; the root-mean-square roughness (RMS) is 1.5–2.5 nm. The granules for PFCN0.5 are somewhat wider and larger than those for FFCN0.5. The tendency to aggregation decreases markedly with an increase in the proportion of dicyano-substituted comonomer. The surface morphology becomes granular-fibrous, which is typical of the films of many polymers that are formed by the spin-coating method. 

### 3.3. Photophysical Properties

The UV–vis absorption spectra of the FFCN and PFCN polymers were measured in chloroform. The absorption maximum of the FFCN and PFCN polymers is 389 nm, which is comparable with polyfluorene and can be attributed to the π−π* transition in the fluorene fragments [12,20]. The same absorption maxima were observed for the FFCN and PFCN polymer films prepared from toluene (Supplementary Materials, Figure S3). The shoulder at ca. 420 nm appeared in the absorption spectra after the heating of the films at 80 °C for 4 h due to the β-phase formation [12,21,22,23].

The photoluminescence spectra of copolyfluorenes in chloroform solution show a well-resolved vibronic structure with bands at 416, 440, and 471 nm assigned to the 0–0, 0–1, and 0–2 band singlet intrachain excitons of the polyfluorene fragment [20,24], respectively. Also, additional bands at 629 and 550 nm were observed in the photoluminescence spectra of the FFCN and PFCN polymers, respectively. 

In the photoluminescence spectra of the copolyfluorene films (Supplementary Materials, Table S2, Figure S4), the most intensive bands, at 550 and 500 nm for FFCN and PFCN, respectively, can be attributed to the incorporation of dicyanostilbene and dicyanophenantrene fragments into the polyfluorene main chain.

Probably, the observed Stokes shifts (Supplementary Materials, Table S2) are connected to the degree of structural reorganization of the two-fold C_2V_-symmetrical luminophoric moiety (dicyanostilbene/dicyanophenanthrene) of the copolymers or intramolecular charge-transfer characteristics (Figure 1) of the copolymers in the film state or intermolecular charge-transfer between the dicyano moieties and fluorene moieties in the copolymers.

### 3.4. OLEDs Based on the Polymers and Their Spectral and Optoelectronic Properties

**Figure 4:**
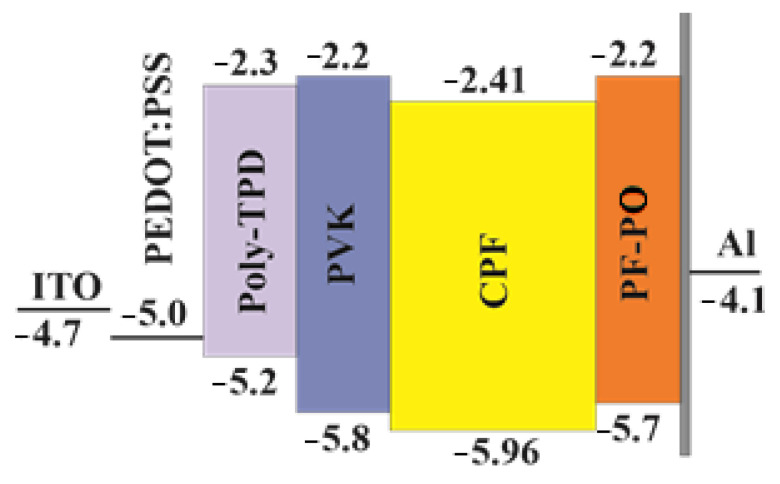
Energy band diagram of OLED structures. Average values of LUMO (−2.41 ± 0.03 eV) and HOMO (5.96 ± 0.03 eV) levels of CPFs according to cyclic voltammetry data are given (Table 2).

The electroluminescent properties of CPFs were studied in OLED structures (type III) ITO/PEDOT-PSS (35 nm)/p-TPD (30 nm)/PVK (5 nm)/EML (70–75 nm)/PF-PO (20 nm)/LiF (1 nm)/Al (80 nm). Here, p-TPD and PVK denote poly (N,N’-bis-4-butylphenyl-N,N’-bisphenyl)benzidine and poly(9-vinylcarbazole), respectively. We used a double hole transport layer (HTL) made of p-TPD/PVK to form a stepwise change in energy levels for efficient hole injection into the CPFs’ emissive layers (EML). Also, as the LUMO of PVK is significantly closer to vacuum compared with that of the CPFs, PVK blocks the flow of electrons from EML (Figure 4). PF-PO (9,9-bis(6-diethoxylphosphorylhexyl)fluorene) [25], here, serves as an electronic transport layer (ETL), where ITO is the anode and LiF/Al is the cathode.

As can be clearly seen from Table 2, the HOMO and LUMO energy levels of the studied CPFs measured using the CVA method do not change significantly with a change in the concentration of additives in the main chain of polyfluorenes and are well matched to the levels of LUMO PF-PO and HOMO PVK, which provides a balanced injection of electrons and holes into the EML (Figure 4). The effect of temperature on the electroluminescent properties of films was studied. It was shown that the electroluminescence practically does not change at 110 °C.

**Figure 5:**
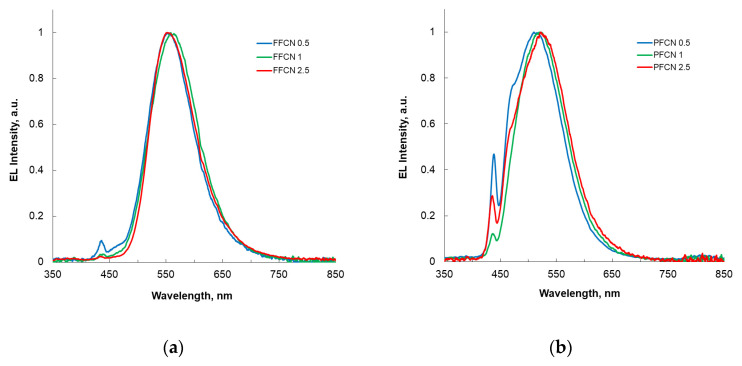
EL spectra of the studied OLEDs based on EML: (**a**) FFCN; (**b**) PFCN series.

**Figure 6:**
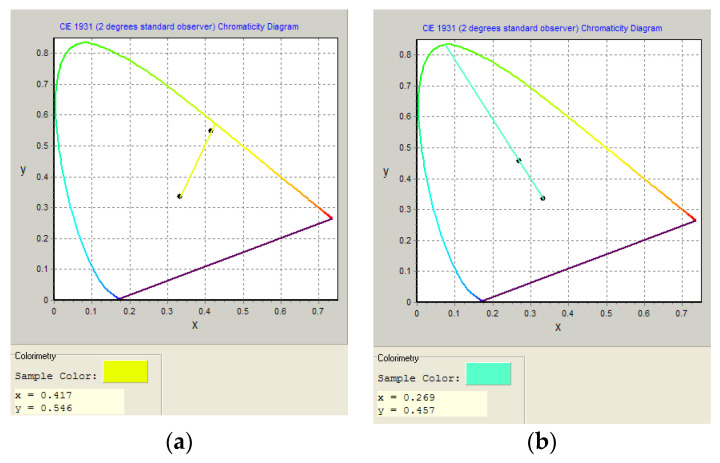
The CIE chromaticity diagrams of PLEDs based on EML: (**a**) FFCN 2.5; (**b**) PFCN 2.5.

In the studied CPFs, the EL bands are broad and located in the entire visible range of the spectrum, and their maxima are shifted by 10–20 nm towards the long-wavelength region relative to the corresponding photoluminescence bands (Table 3). The broad spectra of the EL of the studied polymers will further allow the use of dicyano-comonomers to create copolyfluorenes with white luminescence. The color of the OLED radiation varies from yellow-green (with a maximum EL at 553–562 nm) for the FFCN series to greenish-blue (514–523 nm) for PFCN CPFs (Figure 5 and Figure 6).

The points corresponding to the chromaticity of the radiation of the studied OLEDs do not lie on the border of the locus, which indicates that the color of the radiation is not saturated due to the fact that the FWHM of the EL bands exceeds 100 nm (Figure 6).

The intensity of the EL bands at 435 and 460 nm, characteristic of the fluorene units in the CPFs of the FFCN series, is quite low and decreases with an increasing content of dicyanostilbene additives, which indicates an efficient energy transfer from the fluorene units. For the CPFs of the PFCN series, the fluorine fragments of the polymer chain make a more significant contribution to the EL spectra at a low content of dicyanophenanthrene additives. An increase in the amount of the latter in the polymer chain reduces the contribution of fluorene fragments to the EL spectrum, but the energy transfer process can probably be affected by the tendency to form π-stacking of the dicyanophenanthrene fragments [6,8].

**Figure 7:**
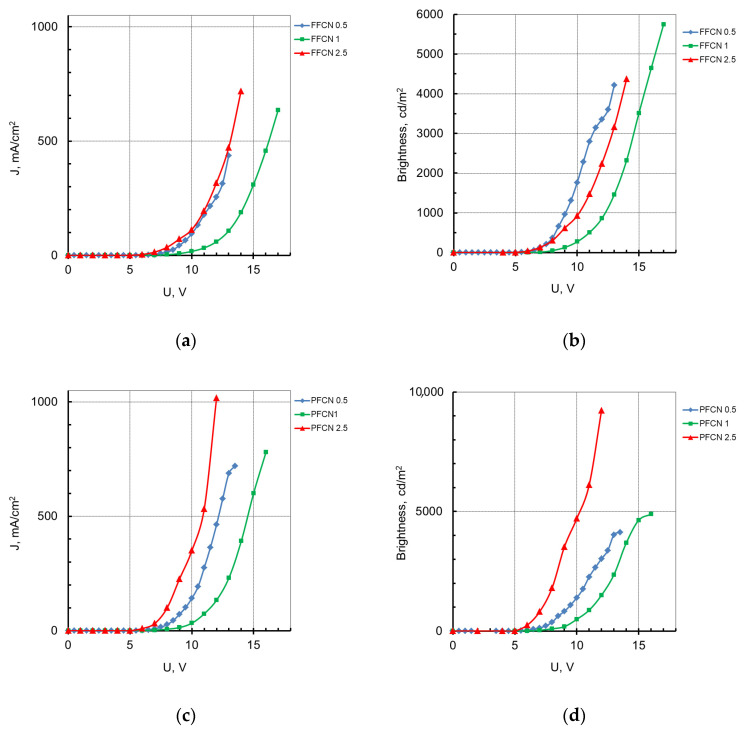
Current-voltage dependence (**a**,**c**) and voltage-brightness characteristics (**b**,**d**) of the OLEDs based on EML with the CPFs of series FFCN and PFCN, respectively.

In PLEDs with the EML based on the CPFs of the FFCN series, the maximum brightness was observed for the sample with FFCN1 and reached 5750 cd/m^2^ (Table 3, Figure 7b), while, for FFCN0.5 and FFCN2.5, it was 4210 and 4370 cd/m^2^, respectively. This behavior may be related to the cis-trans isomerization process of dicyanostilbene additives, which is difficult to control during layer formation and can affect the photoluminescence quantum yield and charge carrier mobility. The dicyanophenanthrene units have a more rigid structure compared to dicyanostilbene, and in the PLED based on them, an increase in maximum brightness is observed with an increase in the content of the additive in the polymer chain. For the PLED based on PFCN2.5, it reaches 9230 cd/m^2^ (Figure 7d) at an applied voltage of 12V, which is the highest value for all CPFs studied in this work. The maximum current efficiency in this case was 3.33 cd/A (Table 3). The EL characteristics of the PLEDs based on the studied CPFs can be affected by the morphology of the polymer films, which requires a separate study. For the OLED structures with the best current efficiency based on PFCN2.5 and FFCN1, the EQE values were estimated, and are 1.4 and 1.7%, respectively.

### 3.5. Cyclic Voltammetry

From the tangents (red lines) to the cyclic voltammogram (CVA) curves of FFCN and PFCN (Supplementary Materials, Figure S5), the oxidation and reduction potentials were calculated and the corresponding data for the HOMO and LUMO levels are listed in Table 2.

### 3.6. Theoretical Calculations

The obtained results of the maxima in the absorption and emission spectra for models 1 and 2, calculated by the TD/SMD/B3LYP/cc-rVDZ method, are presented in Table 1. Considering the maxima in the absorption and emission spectrum, when toluene substituents are added to the phenanthrene molecule, the maximum is shifted from 352 nm to 423 nm, with a significant increase in the oscillator strength. The same pattern is observed when looking at the emission maximum; there is a large shift in the emission wavelength (from 371 to 486 nm) with an increase in the oscillator strength and, consequently, the probability of this process. Calculations show that conjugation is important for such a system and, therefore, model 2, with toluene substituents, is more suitable (Figure 1(b1)). Then, model 2 was used for further calculation of the optical properties (Table 4).

Two functionals, CAM-B3LYP and B3LYP, have been used for model 2. In general, CAM-B3LYP gives results closer to the experiment for charge transfer systems. The results which were obtained (Table 4) show that the maxima in the absorption spectrum shift towards the short wavelength region (359 nm for CAM-B3LYP and 423 nm for B3LYP) while the oscillator strength increases (0.722 for CAM-B3LYP and 0.526 for B3LYP). A similar picture is observed for the emission maximum—the wavelength shifts to the short wavelength region for CAM-B3LYP (from 486 to 417 nm) and the oscillator strength increases (from 0.631 to 0.809). Theoretical calculations show that the charge transfer takes place in the polymer chain in the phenanthrene molecule, so that the CAM-B3LYP functional better describes the experimental data and is more suitable for describing the photophysical properties of this system.

## 4. Conclusions

In summary, the synthesis of copolymers of 9,9-dioctyl-9H-fluorene and low band-gap comonomers, such as α,α’-dicyanostilbene and phenanthrene-9,10-dicarbonitrile, can be accomplished by the Suzuki−Miyaura cross-coupling reaction.

The PL emission spectra of the synthesized polymers show significant energy transfer from fluorene segments to the dicyanostilbene and dicyanophenanthrene units. These data are in good agreement with our theoretical calculations. Our theoretical calculations show that the charge transfer takes place in the polymer chain from fluorene to the phenanthrene moieties.

We were able to tune the emission color in thin films by incorporating small amounts of low band-gap comonomers. These efficient excitonic energy traps play an important role in suppressing the troublesome formation of excimers in thin films of polyfluorene copolymers. The dicyanophenanthrene moieties have a more rigid structure compared to dicyanostilbene, and in PLED based on them, an increase in maximum brightness is observed with an increase in the content of the additive in the polymer chain. The FFCN1 and PFCN2.5 polymers show the best device performances with maximum luminous efficiencies of 5750 cd m^−2^ (1.63 cd A^−1^) and 9230 cd m^−2^ (3.33 cd A^−1^), respectively. The FFCN and PFCN polymers show yellow-green and greenish-blue emissions, respectively, with superior EL stability and high luminance with comparable current efficiency, and thus have great potential for application in light-emitting devices.

## Figures and Tables

**Table 1:** Molecular mass ^1^ characteristics of copolyfluorenes.

CPF Name	Comonomer Loading (x), mol%	M_w_ × 10^−3^ g/mol	M_n_ × 10^−3^ g/mol	M_w_/M_n_
FFCN0.5	0.5	20.5	14.4	1.4
FFCN1	1.0	23.5	16.8	1.4
FFCN2.5	2.5	15.5	12.2	1.3
PFCN0.5	0.5	32.5	20.9	1.55
PFCN1	1.0	20.2	13.5	1.5
PFCN2.5	2.5	11.5	9.0	1.3

^1^ LS detector data presented.

**Table 2:** LUMO and HOMO levels (eV) determined from the CV data.

Material	LUMO	HOMO	E_LUMO-HOMO_
FFCN 0.5	−2.39	−5.96	3.57
FFCN 1	−2.44	−5.99	3.55
FFCN 2.5	−2.44	−5.98	3.54
PFCN 0.5	−2.38	−5.95	3.57
PFCN 1	−2.39	−5.93	3.54
PFCN 2.5	−2.44	−5.94	3.50

**Table 3:** EL characteristics of the studied PLEDs with different light-emitting layers.

Ligt-Emitting Layer	U_on_, V	Max. Brightness, cd/m^2^	Max. Efficiency		CIE		λ_max_ EL, nm
			CE, cd/A	PE, lm/W	x	y	
FFCN 0.5	4.4	4210	3.67	2.33	0.395	0.534	436, 462, 554
FFCN1	6.0	5750	1.63	0.62	0.420	0.533	437, 562
FFCN 2.5	4.2	4370	0.97	0.61	0.417	0.546	435, 553
PFCN 0.5	4.4	4120	1.96	1.24	0.234	0.420	438, 472, 514
PFCN1	5.1	4880	1.48	0.54	0.267	0.495	436, 520
PFCN 2.5	4.0	9230	3.33	1.33	0.269	0.457	436, 470, 523

**Table 4:** Absorption and emission maxima for models 1 and 2 calculated by the B3LYP functional and absorption and emission maxima for model 2 calculated by CAM-B3LYP and B3LYP functional.

**State**	**Model 1 and 2 According to Figure 1. TD/SMD/B3LYP/cc-pVDZ**							
	**Absorption**				**Emmision**			
	**Model 1**		**Model 2**		**Model 1**		**Model 2**	
	**λ**	***f***	**λ**	***f***	**λ**	***f***	**λ**	***f***
S_0_ → S_1_	369	0.045	423	0.526	371	0.262	486	0.631
S_0_ → S_2_	352	0.292	375	0.171				
Energy of molecular orbitals, eV								
	HOMO	LUMO	HOMO	LUMO				
E_MO_	−6.54	−2.64	−6.00	−2.66				
E_LUMO-HOMO_	3.91		3.34					
**State**	**Model 2. TD/SMD/CAM-B3LYP/cc-pVDZ and TD/SMD/B3LYP/cc-pVDZ**							
	**Absorption**				**Emmision**			
	**CAM-B3LYP**		**B3LYP**		**CAM-B3LYP**		**B3LYP**	
	**λ**	***f***	**λ**	***f***	**λ**	***f***	**λ**	***f***
S_0_ → S_1_	359	0.722	423	0.526	417	0.809	486	0.631
S_0_ → S_2_	332	0.108	375	0.171				
Energy of molecular orbitals, eV								
	HOMO	LUMO	HOMO	LUMO				
E_MO_	−7.31	−1.71	−6.00	−2.66				
E_LUMO-HOMO_	5.60		3.34					

λ and *f* are the absorption wavelength (nm) and oscillator strength of the S_0_ → S_1_ transition, respectively.

## Data Availability

The data presented in this study are available on request from the corresponding author.

## Supplementary Materials

The following supporting information can be downloaded at: https://www.mdpi.com/article/10.3390/ma16165592/s1. Table S1: FT-IR and ^1^H NMR selected data for synthesized copolyfluorenes. Table S2: Spectral data for CPFs films after heating for 4h at 80 °C. Figure S1: ^1^H NMR spectra of CPFs in CDCl_3_. Figure S2: FT-IR spectra of synthesized CPFs. Figure S3: UV-vis spectra of FFCN2.5 and PFCN2.5 films before (black line) and after heating at 80 °C (red line). Figure S4: Luminescence spectra of CPFs films (λ_ex_ = 385 nm). Figure S5: CVA curves for CPFs.
