# Supplementary material for: Synthesis, Photo- and Electroluminescence of New Polyfluorene Copolymers Containing Dicyanostilbene and 9,10-Dicyanophenanthrene in the Main Chain
Source: Materials (Basel). 2023 Aug 12;16(16):5592. doi: 10.3390/ma16165592 (PMC10456789; doi:10.3390/ma16165592)
Supplement: Supplementary file 1 [file materials-16-05592-s001.zip › materials-2540697-supplementary.pdf]

Supporting Information

Synthesis, Photo- and Electroluminescence of New Polyfluorene Copolymers Containing Dicyanostilbene and 9,10-Dicyanophenanthrene in the Main Chain

Anton A. Yakimanskiy [1], Ksenia I. Kaskevich [1], Elena V. Zhukova [1], Ivan A. Berezin [1], Larisa S. Litvinova [1], Tatiana G. Chulkova [1], Dmitriy A. Lypenko [2], Artem V. Dmitriev [2], Sergey I. Pozin [2], Natalia V. Nekrasova [2], Felix N. Tomilin [3,4], Daria A. Ivanova [4] and Alexander V. Yakimansky [1,*]

[1] Institute of Macromolecular Compounds RAS, Bolshoi Prospect of Vasilyevsky Island 31, St. Petersburg 199004, Russia; yakimanskii@gmail.com (A.A.Y.); kaskevich-ksenia@yandex.ru (K.I.K.); zukovaev@mail.ru (E.V.Z.); bereziv@mail.ru (I.A.B.); larissa_litvinova@hotmail.com (L.S.L.); tgc@mail.ru (T.G.C.)

[2] A.N. Frumkin Institute of Physical Chemistry and Electrochemistry RAS, Leninskiy Prospect 31, bld.4, Moscow 119071, Russia; dalypenko@gmail.com (D.A.L.); oleduff@mail.ru (A.V.D.); sergip74@gmail.com (S.I.P.); natalianek@yandex.ru (N.V.N.)

[3] Kirensky Institute of Physics, Federal Research Center KSC SB RAS, Krasnoyarsk 660036, Russia; felixnt@gmail.com

[4] School of Non-Ferrous Metals and Material Science, Siberian Federal University, Krasnoyarsk 660041, Russia; daha-ha@mail.ru

* Correspondence: yakimansky@yahoo.com; Tel.: +7-812-3237407

Table S1. FT-IR and ^{1}H NMR selected data for synthesized copolyfluorenes.

**CPFs name**	**IR data, cm^{-1}**	**^{1}H NMR data, ppm**
FFCN0.5	2921, s (ν_{as} CH_2)<br>2852, s (ν_{s} CH_2)<br>1454, s (ν_{s} $C\text{-}C_{ar}$)<br>812, s (δ CH_{ar})	7.88-7.86 (m, 2H), 7.74-7.70 (m, 4H), H_{ar} fluorene<br>4.14 (q, *J* = 7.0 Hz), $CH_3C\boldsymbol{H}_2O$- (terminal)<br>2.5-1.75 (m, 4H) CH_2 octyl<br>1.49 (t, *J* = 7.0 Hz) $C\boldsymbol{H}_3CH_2O$- (terminal)<br>1.25-1.17 (m, 22H), CH_2 octyl<br>0.86-0.83 (m, 10H), CH_2 and CH_3 octyl
FFCN1	2919, s (ν_{as} CH_2)<br>2853, s (ν_{s} CH_2)<br>1452, s (ν_{s} $C\text{-}C_{ar}$)<br>811, s (δ CH_{ar})	7.88-7.86 (m, 2H), 7.74-7.70 (m, 4H), H_{ar} fluorene<br>4.14 (q, *J* = 7.0 Hz), $CH_3C\boldsymbol{H}_2O$- (terminal)<br>2.5-1.75 (m, 4H) CH_2 octyl<br>1.49 (t, *J* = 7.0 Hz) $C\boldsymbol{H}_3CH_2O$- (terminal)<br>1.25-1.17 (m, 22H), CH_2 octyl<br>0.86-0.83 (m, 10H), CH_2 and CH_3 octyl
FFCN2.5	2920, s (ν_{as} CH_2)<br>2853, s (ν_{s} CH_2)<br>1454, s (ν_{s} $C\text{-}C_{ar}$)<br>812, s (δ CH_{ar})	7.88-7.86 (m, 2H), 7.74-7.70 (m, 4H), H_{ar} fluorene<br>4.14 (q, *J* = 7.0 Hz), $CH_3C\boldsymbol{H}_2O$- (terminal)<br>2.5-1.75 (m, 4H) CH_2 octyl<br>1.49 (t, *J* = 7.0 Hz) $C\boldsymbol{H}_3CH_2O$- (terminal)<br>1.25-1.17 (m, 22H), CH_2 octyl<br>0.86-0.83 (m, 10H), CH_2 and CH_3 octyl
PFCN0.5	2918, s (ν_{as} CH_2)<br>2853, s (ν_{s} CH_2)<br>1453, s (ν_{s} $C\text{-}C_{ar}$)<br>810, s (δ CH_{ar})	7.88-7.86 (m, 2H), 7.74-7.70 (m, 4H), H_{ar} fluorene<br>4.14 (q, *J* = 7.0 Hz), $CH_3C\boldsymbol{H}_2O$- (terminal)<br>2.5-1.75 (m, 4H) CH_2 octyl<br>1.49 (t, *J* = 7.0 Hz) $C\boldsymbol{H}_3CH_2O$- (terminal)<br>1.25-1.17 (m, 22H), CH_2 octyl<br>0.86-0.83 (m, 10H), CH_2 and CH_3 octyl
PFCN1	2918, s (ν_{as} CH_2)<br>2854, s (ν_{s} CH_2)<br>1453, s (ν_{s} $C\text{-}C_{ar}$)<br>812, s (δ CH_{ar})	7.88-7.86 (m, 2H), 7.74-7.70 (m, 4H), H_{ar} fluorene<br>4.14 (q, *J* = 7.0 Hz), $CH_3C\boldsymbol{H}_2O$- (terminal)<br>2.5-1.75 (m, 4H) CH_2 octyl<br>1.49 (t, *J* = 7.0 Hz) $C\boldsymbol{H}_3CH_2O$- (terminal)<br>1.25-1.17 (m, 22H), CH_2 octyl<br>0.86-0.83 (m, 10H), CH_2 and CH_3 octyl
PFCN2.5	2918, s (ν_{as} CH_2)<br>2854, s (ν_{s} CH_2)<br>1450, s (ν_{s} $C\text{-}C_{ar}$)<br>811, s (δ CH_{ar})	7.88-7.86 (m, 2H), 7.74-7.70 (m, 4H), H_{ar} fluorene<br>4.14 (q, *J* = 7.0 Hz), $CH_3C\boldsymbol{H}_2O$- (terminal)<br>2.5-1.75 (m, 4H) CH_2 octyl<br>1.49 (t, *J* = 7.0 Hz) $C\boldsymbol{H}_3CH_2O$- (terminal)<br>1.25-1.17 (m, 22H), CH_2 octyl<br>0.86-0.83 (m, 10H), CH_2 and CH_3 octyl

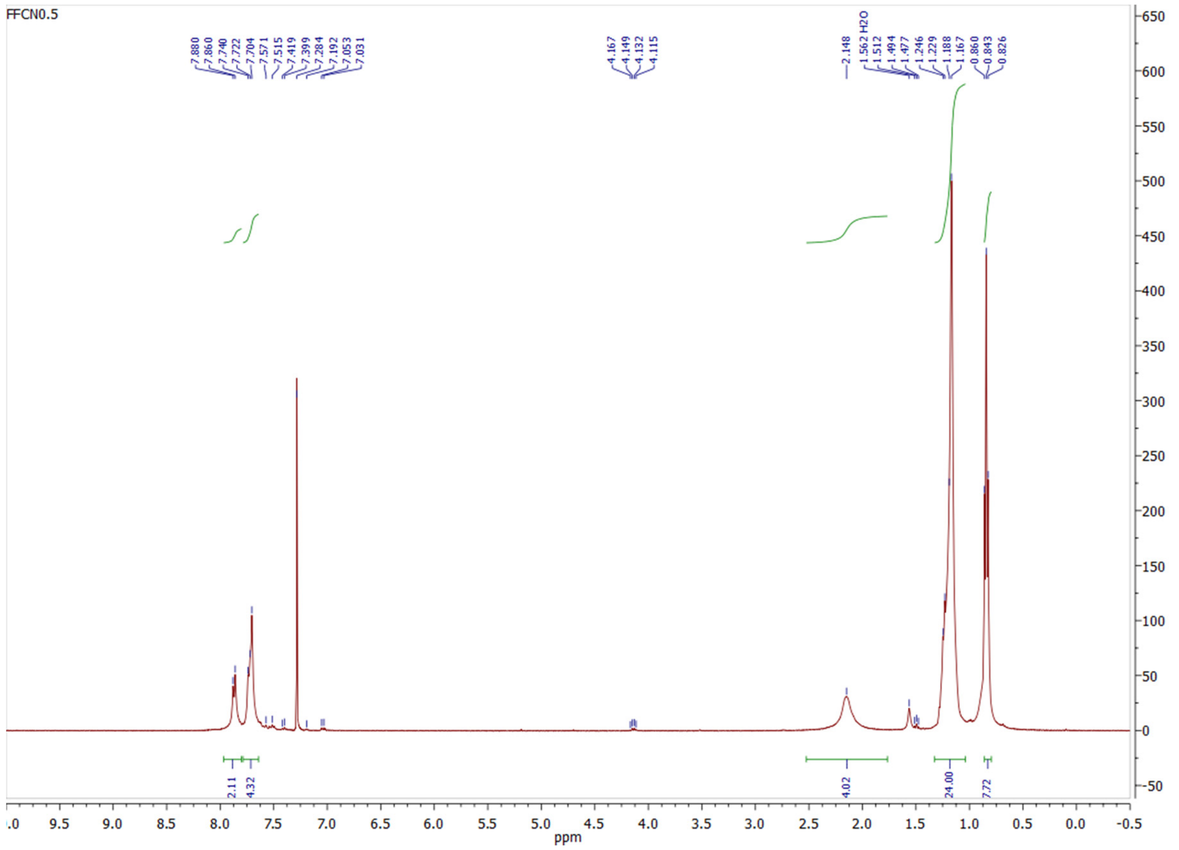
FFCN0.5
7.880 7.860 7.740 7.722 7.704 7.571 7.515 7.419 7.399 7.284 7.192 7.053 7.031
4.167 4.149 4.132 4.115
2.148 1.562 H2O 1.512 1.494 1.477 1.246 1.229 1.188 1.167 0.860 0.843 0.826
2.11 4.32 4.02 24.00 7.72
ppm

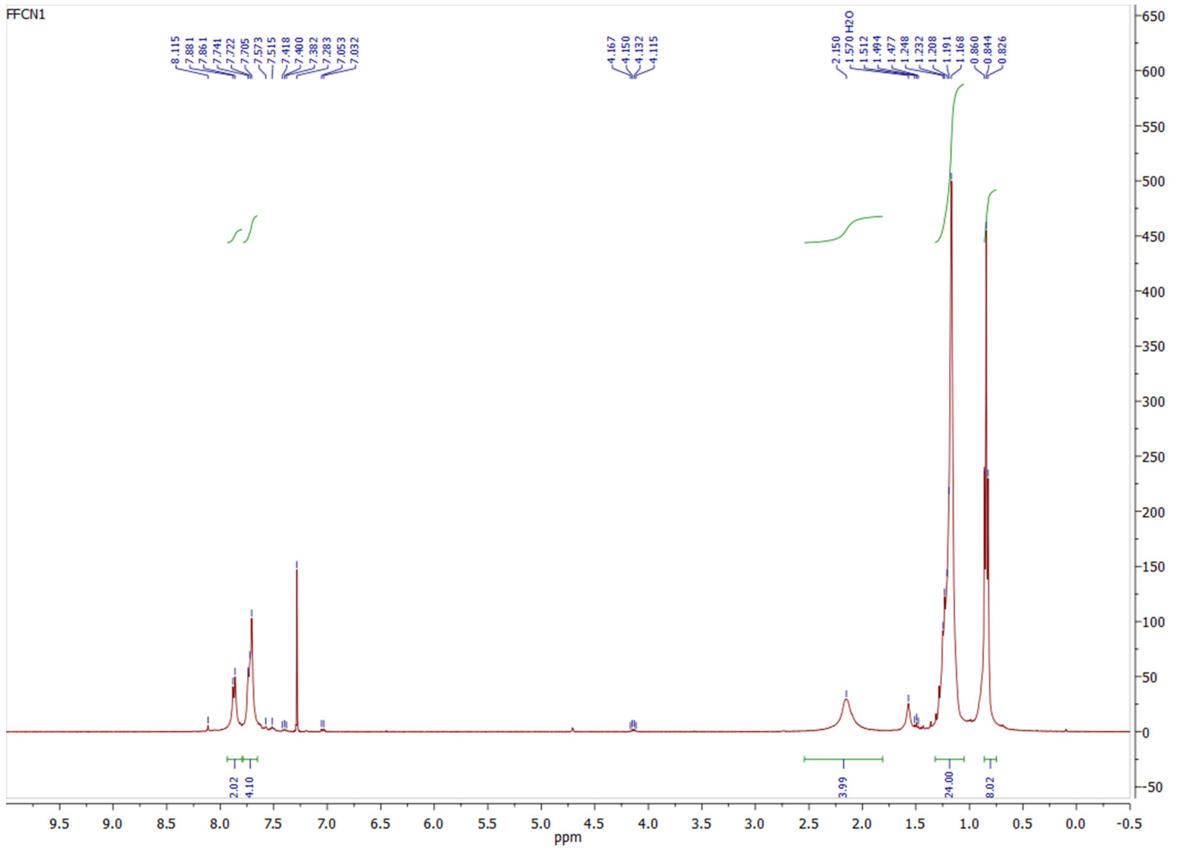
FFCN1
8.115 7.881 7.861 7.741 7.722 7.705 7.573 7.515 7.418 7.400 7.382 7.283 7.053 7.032
4.167 4.150 4.132 4.115
2.150 1.570 H2O 1.512 1.494 1.477 1.248 1.232 1.208 1.191 1.168 0.860 0.844 0.826
2.02 4.10 3.99 24.00 8.02
ppm

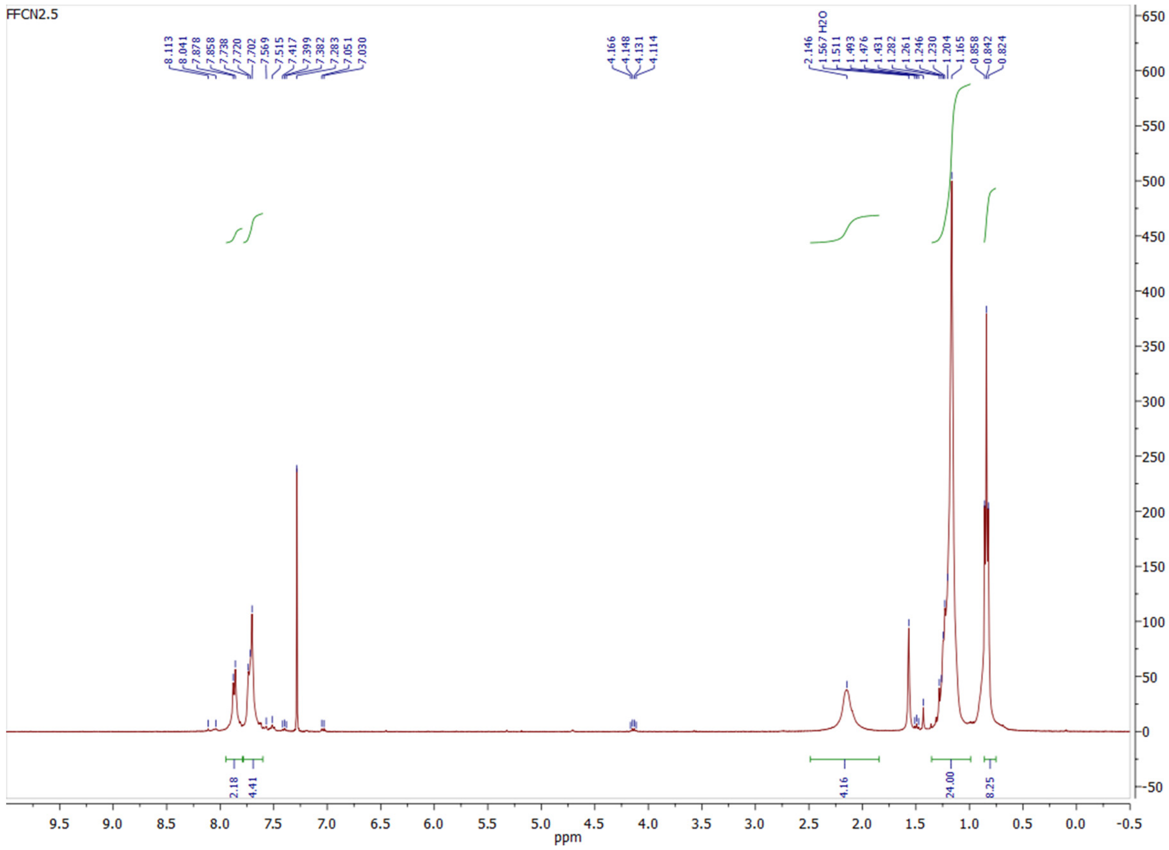
FFCN2.5
8.113 8.041 7.878 7.858 7.738 7.720 7.702 7.569 7.515 7.417 7.399 7.382 7.283 7.051 7.030
4.166 4.148 4.131 4.114
2.146 1.567 H2O 1.511 1.493 1.476 1.431 1.282 1.261 1.246 1.230 1.204 1.165 0.858 0.842 0.824
2.18 4.41 4.16 24.00 8.25
ppm

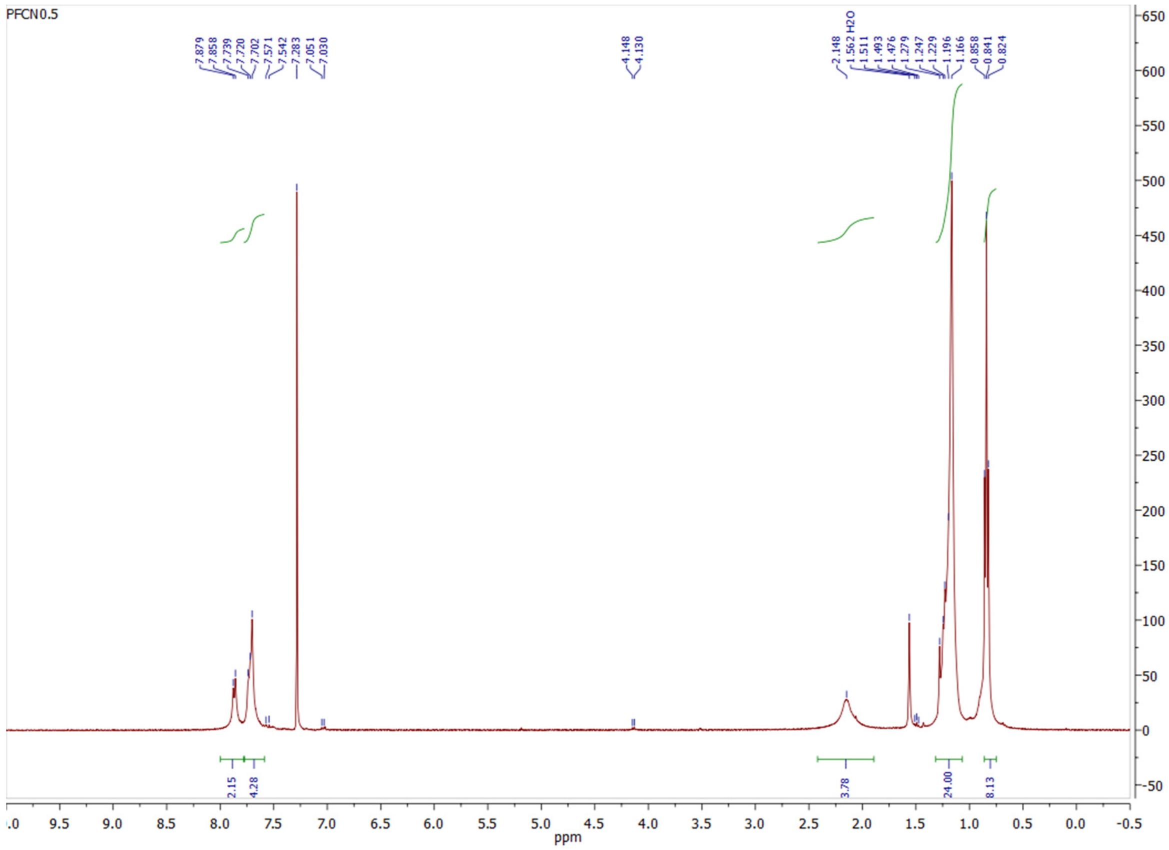
PFCN0.5
7.879 7.858 7.739 7.720 7.702 7.571 7.542 7.283 7.051 7.030
4.148 4.130
2.148 1.562 H2O 1.511 1.493 1.476 1.279 1.247 1.229 1.196 1.166 0.858 0.841 0.824
2.15 4.28 3.78 24.00 8.13
ppm

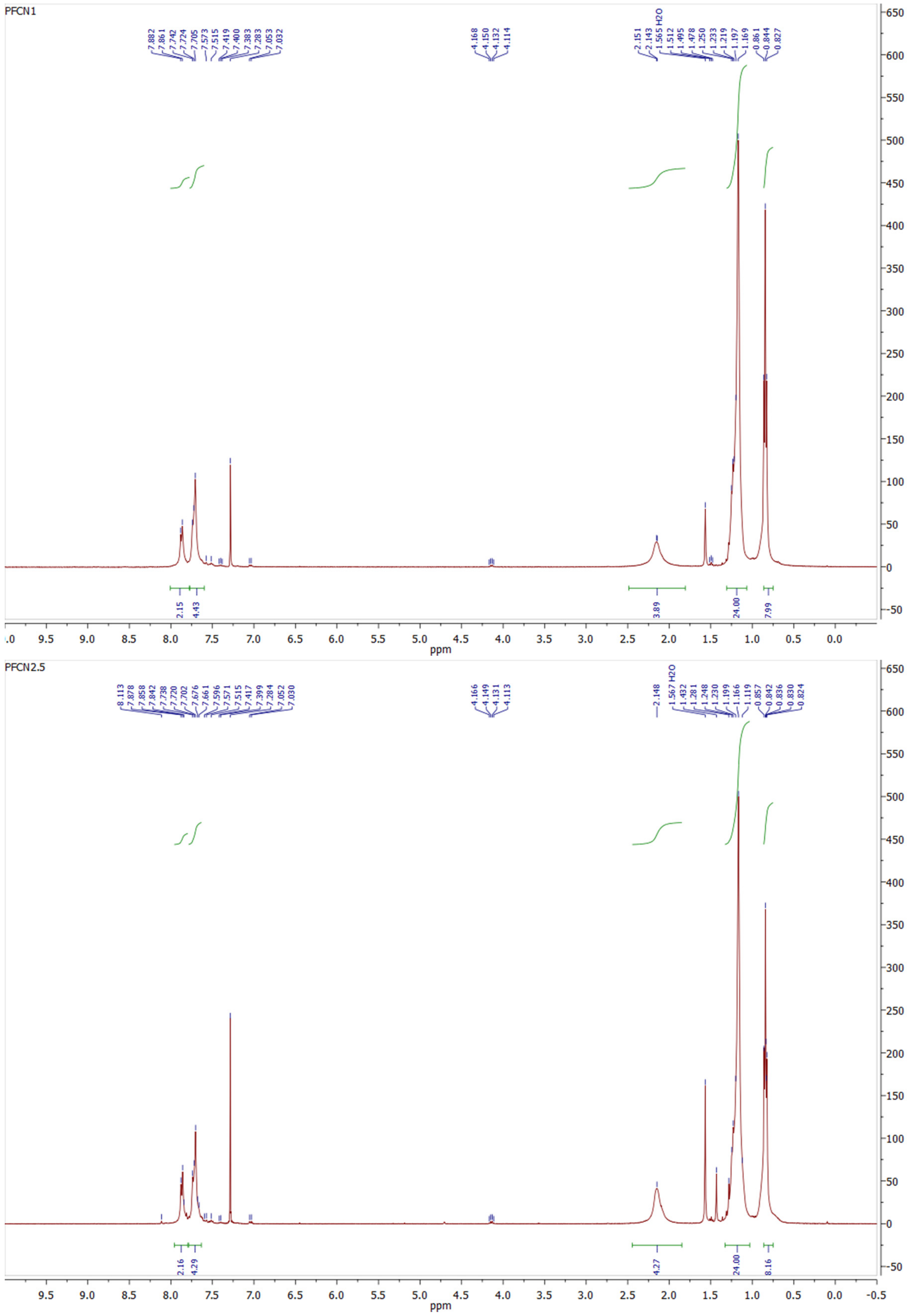

Figure S1. ^{1}H NMR spectra of CPFs in $CDCl_3$.

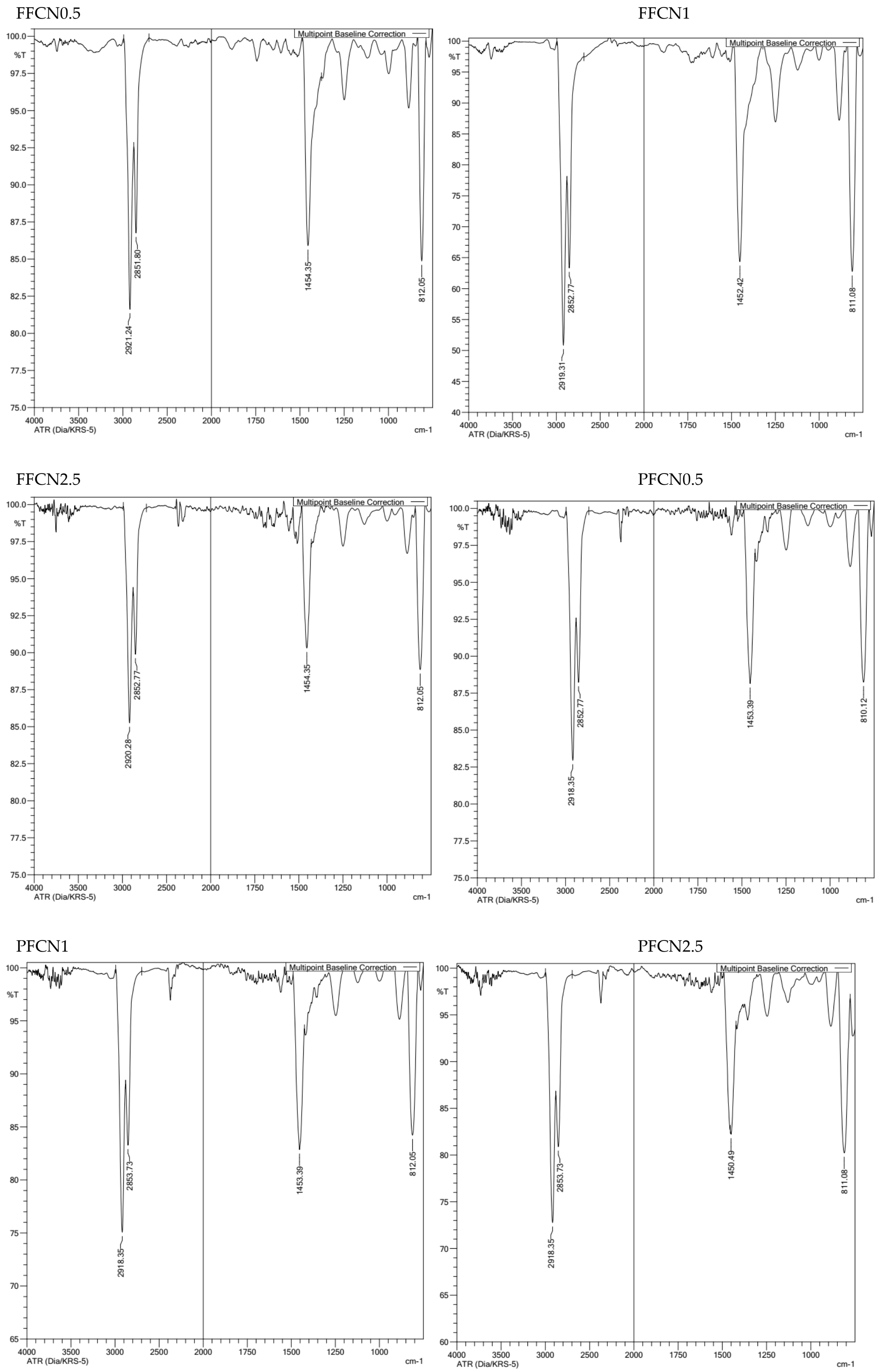

Figure S2. FT-IR spectra of synthesized CPFs.

Table S2. Spectral data for CPFs films after heating for 4h at 80°C.

CPFs name	λ_{abs}, nm	λ_{em}, nm	Stokes shift, nm/cm^{-1}/eV
FFCN0.5	385, 424*	542	118/5135/0.64
FFCN1	385, 426*	545	119/5126/0.64
FFCN2.5	382, 422*	551	129/5548/0.69
PFCN0.5	385, 426*	499	73/3434/0.43
PFCN1	385, 426*	505	79/3672/0.46
PFCN2.5	385, 424*	463	39/1987/0.25

* Shoulder

FFCN2.5 PFCN2.5

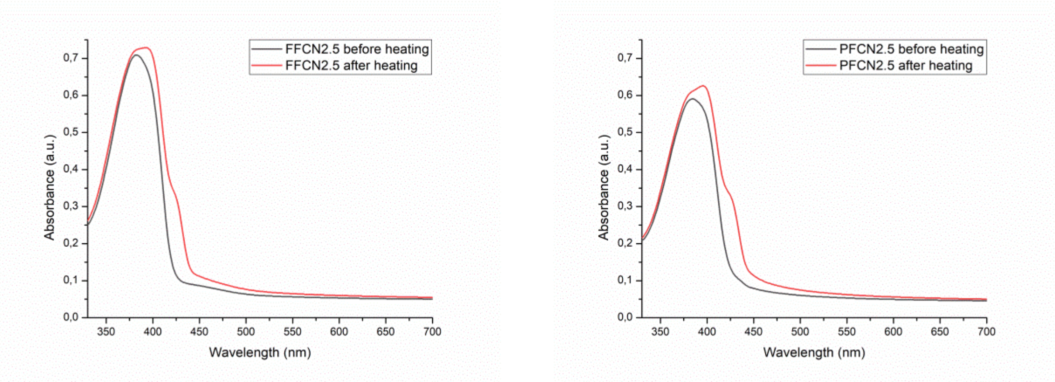

Figure S3. UV-vis spectra of FFCN2.5 and PFCN2.5 films before (black line) and after heating at 80°C (red line).

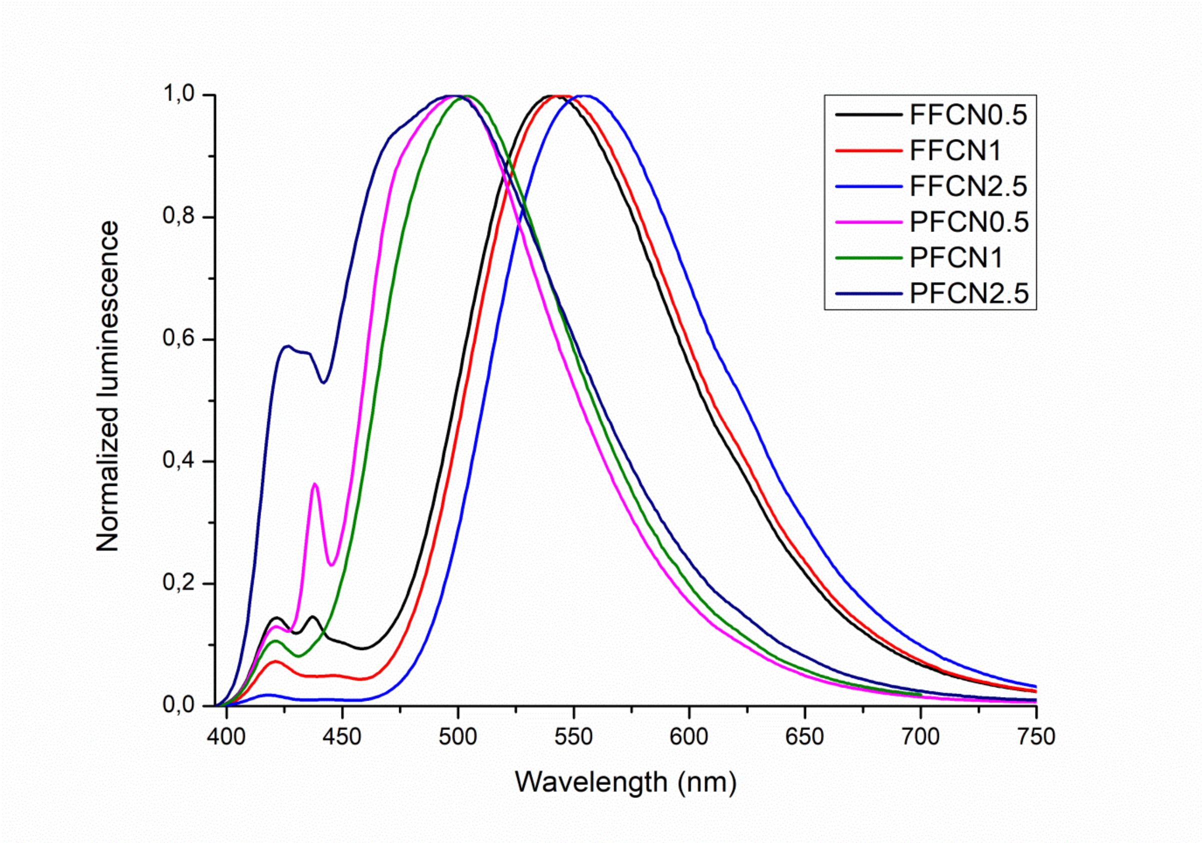

Figure S4. Luminescence spectra of CPFs films (λ_{ex} = 385 nm).

Cyclic Voltammetry

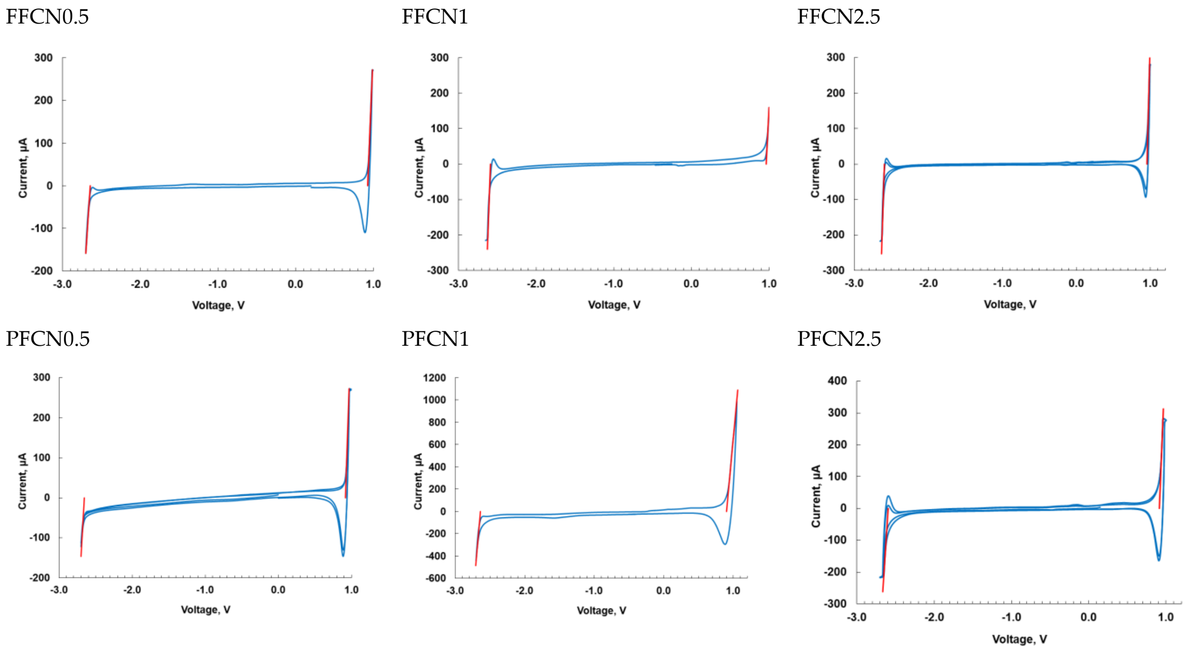

Figure S5. CVA curves for CPFs.
